# Age Similarity in Emotion Perception Based on Eye Gaze Manipulation

**DOI:** 10.1093/geroni/igaa057.1474

**Published:** 2020-12-16

**Authors:** Yosra Abualula, Eric Allard

**Affiliations:** 1 Cleveland State University, Lakewood, Ohio, United States; 2 Cleveland State University, Cleveland, Ohio, United States

## Abstract

The purpose of this study was to examine age differences in emotion perception as a function of emotion type and gaze direction. Old and young adult participants were presented with facial images showing happiness, sadness, fear, anger and disgust while having their eyes tracked. The image stimuli included a manipulation of eye gaze. Half of the facial expressions had a directed eye gaze while the other half showed an averted gaze. A 2 (age) x 2 (gaze) x 5 (emotion) repeated measures ANOVA was used to analyze emotion perception scores and fixation to eye and mouth regions of the face. The manipulation of eye gaze yielded more age similarities than differences in emotion perception. Overall, we did not detect age differences in recognition ability. However, we found that certain emotion categories differentially impacted emotion perception. Interestingly, we observed that an averted gaze led to beneficial performance for fear and disgust faces. Additionally, participants spent more time fixating on the eye regions of sad facial expressions. We discuss how naturalistic manipulations of various facial features could impact age-related differences (or similarities) in emotion perception.

